# Changes in Physical Fitness, Bone Mineral Density and Body Composition During Inpatient Treatment of Underweight and Normal Weight Females with Longstanding Eating Disorders

**DOI:** 10.3390/ijerph9010315

**Published:** 2012-01-19

**Authors:** Solfrid Bratland-Sanda, Egil W. Martinsen, Jorunn Sundgot-Borgen

**Affiliations:** 1 Department of Sport and Outdoor Life Science, Telemark University College, Hallvard Eikas Plass, 3800 Bø i Telemark, Norway; 2 Research Institute, Modum Bad Psychiatric Center, Badeveien, 3370 Vikersund, Norway; 3 Department of Mental Health and Addiction, Oslo University Hospital, Pb 4956 Nydalen, 0424 Oslo, Norway; Email: e.w.martinsen@medisin.uio.no; 4 Department of Clinical Medicine, University of Oslo, Pb 1039 Blindern, 0315 Oslo, Norway; 5 Department of Sports Medicine, Norwegian School of Sport Sciences, Pb 4014 Ullevål Stadion, 0806 Oslo, Norway; Email: j.s.borgen@nih.no

**Keywords:** eating disorders, psychiatry, females, adult, exercise, VO_2max_, resistance training

## Abstract

The purpose of this study was to examine changes in aerobic fitness, muscular strength, bone mineral density (BMD) and body composition during inpatient treatment of underweight and normal weight patients with longstanding eating disorders (ED). Twenty-nine underweight (BMI < 18.5, n = 7) and normal weight (BMI ≥ 18.5, n = 22) inpatients (mean (SD) age: 31.0 (9.0) years, ED duration: 14.9 (8.8) years, duration of treatment: 16.6 (5.5) weeks) completed this prospective naturalistic study. The treatment consisted of nutritional counseling, and 2 × 60 min weekly moderate intensive physical activity in addition to psychotherapy and milieu therapy. Underweight patients aimed to increase body weight with 0.5 kg/week until the weight gain goal was reached. Aerobic fitness, muscular strength, BMD and body composition were measured at admission and discharge. Results showed an increase in mean muscular strength, total body mass, fat mass, and body fat percentage, but not aerobic capacity, among both underweight and normal weight patients. Lumbar spine BMD increased among the underweight patients, no changes were observed in BMD among the normal weight patients. Three out of seven underweight patients were still underweight at discharge, and only three out of nine patients with excessive body fat (*i.e.*, >33%) managed to reduce body fat to normal values during treatment. These results calls for a more individualized treatment approach to achieve a more optimal body composition among both underweight and normal to overweight patients with longstanding ED.

## 1. Introduction

Eating disorders (ED) are serious mental illnesses which are also characterized by detrimental somatic changes such as reduced physical fitness, altered body weight and body fat percentage, and poor bone health compared to healthy controls [1]. These impairments occur despite higher physical activity levels among patients compared to controls [2,3]. Physical activity is useful for improvement of both physical fitness, bone mineral density (BMD) and body composition [4], but excessive amounts of physical activity can have opposite effects [5,6]. In a public health perspective, physical fitness, especially aerobic fitness, is one of the most important predictors for good health and prevention of lifestyle related diseases [7]. Although focus on physical changes during treatment of ED is often limited to bone health and body composition, the importance of physical fitness for somatic and mental health calls for studies which also address this issue. 

Despite higher amounts of physical activity among patients with longstanding ED compared to controls, we found no increase in aerobic capacity and reduced muscular strength among patients across the three DSM-IV ED diagnoses anorexia nervosa (AN), bulimia nervosa (BN) and ED not otherwise specified (EDNOS) [2,8]. This fits well with previous studies on patients with AN [9,10,11]. During weight restoration of adolescents with AN, Waller *et al.* [12] and Rigaud *et al.* [13] found only small improvements in aerobic capacity after eight weeks, whereas Fohlin *et al.* [14] found aerobic capacity to increase into normal values. Treatment approaches including regular, systematic physical activity have been found effective in increasing aerobic capacity among patients with AN [15] and BN [16]. Less is known about changes in muscular strength during treatment. Rigaud *et al.* [13] found great improvements in muscular strength on a bicycle ergometer exercise test after 45 days of refeeding in patients with AN. These improvements occurred despite unaltered muscle mass and aerobic capacity. Studies using light-to-moderate intensity resistance training as a part of treatment are inconclusive regarding the effects on muscular strength. Chantler *et al.* [17] found an effect after eight weeks of resistance training, whereas del Valle *et al.* [18] found no effects after three months of resistance training. Although light-to-moderate intensity, the doses of resistance training were different in these two studies, and it is possible that the lack of improvements in Del Valle *et al.* [18] can be explained by an inadequate dose of resistance training. To our knowledge, no studies have examined the changes in aerobic capacity and muscular strength among both underweight and normal weight adult patients across the three diagnoses AN, BN and EDNOS.

Several studies have reported low values of bone mineral density (BMD), especially in the lumbar spine and femur neck, among patients with AN compared to controls [19]. In patients with BN, the literature is inconsistent. Sundgot-Borgen *et al.* [20] found normal BMD values among both patients with BN and healthy controls, whereas others have found low BMD values among both AN and BN patients [21,22,23]. Possible explanations for this inconsistency can be differences in the patients’ previous ED, menstrual irregularities and exercise history. Studies have found a history of AN, especially with adolescent onset, to be a strong predictor of low BMD among patients with BN [21]. In addition, Bratland-Sanda *et al.* [8] found both current and previous high mechanical loading physical activity, but not general weight-bearing physical activity, to be correlated with BMD in lumbar spine, femur neck and total body. Treatment for low BMD values in patients with ED includes pharmaceutical and nutritional strategies, but today there is lack of knowledge regarding the effectiveness of these strategies [24]. Studies following changes in BMD during treatment have found that weight restoration and menstrual recovery are the most important factors in patients with AN [25,26]. To our knowledge, only one prospective study has examined changes in BMD in patients with BN, and in this study only one patient had osteopenia (*i.e.*, t-score, from −1.0 to −2.5 SD) at baseline [27]. Therefore, it is still unclear how low BMD values changes following treatment in both underweight and normal weight patients with ED. 

Body composition measurements separate between fat mass and fat free/lean body mass. The fat mass consists of essential fat mass (*i.e.*, fat mass necessary to maintain normal physiological and cognitive functioning) and storage fat [28]. Behnke developed a theoretical limit for minimal body mass, which states that females have approximately 12% essential body fat [28]. If the body fat percentage drops below this limit, there is a high risk of menstrual irregularities and impaired physiological and cognitive functions [28]. The major weight related public health issue in the western world is obesity, but there is no consensus of when body fat percentage exceeds healthy limits. Several suggest 30–35% to be the upper limit for healthy body fat percentage [28,29,30]. Studies have found reduced brain volume and altered brain function and metabolism among very low weight patients with AN [31,32]. Although these changes can be reversible, abnormalities in brain tissue volume, brain structure and cognitive function have been seen in females with a history of AN [32,33]. Weight restoration is therefore important; however current clinical practice often does not take body composition into account. Existing studies have found weight restoration in patients with AN to consist mainly of increase in fat mass [12,34]. As an immediate effect, Mayer *et al.* [34] found an altered body fat distribution with more visceral adipose tissue, however the body fat distribution was normalized within one year after weight restoration [35]. Several studies have suggested that restrictions regarding physical activity is necessary to achieve weight restoration [36,37,38], whereas Touyz *et al.* [39] did not find anaerobic exercise (*i.e.*, strength training) to interfere with weight gain during treatment of AN patients. Only one study has to our knowledge examined changes in body composition during treatment in patients with BN. Sundgot-Borgen *et al.* [16] found larger enhancement of body composition in patients who exercised compared to patients who received nutritional counseling or cognitive-behavioral therapy. 

Total body weight and body mass index (BMI) are the most common measures of weight restoration. However, BMI and body composition are not necessarily comparable. On one hand, persons with a BMI in the underweight category (*i.e.*, <18.5 kg/m^2^) can have body fat percentage values within normal range, and persons with BMI in the normal weight or overweight category can actually be low on body fat (*i.e.*, certain athlete populations) [28]. Since the majority of patients with ED are normal weight, possible unhealthy body composition in these patients is not necessarily detected and managed in treatment. In most studies concerning physical fitness, BMD and body composition in patients with ED, the samples consist of adolescents with AN. Therefore it is a need for studies covering adult underweight and normal weight patients across the three diagnoses AN, BN and EDNOS. 

### 1.1. Aim of the Study

The aim of this study was to observe changes in physical fitness, BMD and body composition during an inpatient treatment period for underweight and normal weight patients with longstanding eating disorders. In addition, we aimed to explore correlations between changes in body composition and changes in ED psychopathology.

## 2. Methods

### 2.1. Sample

This sample consisted of adult females with longstanding ED, admitted to inpatient treatment at Department of Eating Disorders, Modum Bad Psychiatric Center in Norway. Admission criteria for receiving treatment at this department were meeting the DSM-IV criteria for AN, BN or EDNOS assessed by the Eating Disorders Examination (EDE) interview, inadequate response to previous treatment, age ≥ 18, no serious somatic complications and body mass index (BMI) above 14.5. Exclusion criteria were not being able to perform physical fitness tests, and drop-out from treatment. Of the 65 consecutive patients admitted between January 2006 and July 2007, 55 were eligible. Eleven of these were excluded, due to not being able to perform the physical fitness tests in the study (somatic illness and injury, n = 9, pregnancy, n = 1, and not terminated VO_2max_, n = 1). Fifteen patients dropped out of treatment or withdrew from the study from admission to discharge. The final sample consisted of 29 patients. The study was approved by the Regional Committee for Medical and Health Research Ethics in Southern Norway, and by the Norwegian Social Science Data Services. 

### 2.2. Design

The study had a prospective naturalistic design, and the researchers did not interfere with the treatment. We assessed physical fitness, BMD and body composition at admission and discharge. The treatment was multi-component and lasted 12 to 24 weeks. The main focus of treatment was to reduce symptoms of ED, and to normalize eating behavior through a minimum of four meals per day. The nutritional plan followed the Norwegian recommendation for composition of meals, with approximately 55% carbohydrates, 30% fat and 15% protein [40]. In addition, patients with low BMD values (*i.e.*, >−1 SD z-score obtained by DXA) received Ca^2+^ and vitamin D supplements. The underweight patients committed to gain 0.5 kg/week until their weight gain goal was reached.

Two weekly 60-minute physical activity (PA) moderate intensity group sessions were an obligatory part of the treatment for all patients regardless of body weight. Exclusion criteria for participations in the PA sessions were abnormal electrolyte values, dehydration, and inadequate energy intake during treatment. Apart from this, both underweight and normal weight patients participate in these sessions. The types of activities varied from ball games, such as volleyball and soccer, walking and Nordic walking to strength exercises and horseback riding. The sessions were led by an experienced exercise physiologist, and milieu staff was also present. The treatment also consisted of individual and group psychotherapy, psycho-education and art therapy sessions. More thorough description of the treatment program has been published elsewhere [41].

### 2.3. Assessments

#### 2.3.1. ED Psychopathology

The semi-structured clinical interview Eating Disorders Examination (EDE) version 12.0 [42] was used to classify DSM-IV diagnoses AN, BN and EDNOS. The interviews were performed by highly experienced clinicians independent of the research group. In addition the patients completed the self-report instrument Eating Disorders Inventory (EDI) version 2 [43]. 

#### 2.3.2. Physical Activity

Weekly physical activity was assessed both objectively using accelerometer and through self-report for seven consecutive days at admission. These assessments are more thoroughly described in Bratland-Sanda *et al.* [2]. In addition, questions from the EDE interview were used to determine physical activity amount during the last four weeks prior to admission.

#### 2.3.3. Aerobic Capacity

VO_2max_ was measured using Bruce protocol on a treadmill (Woodway ELG 2, Woodway, Germany). The participants had a five-minute warm up with walking at light to moderate intensity; this warm up was also used to make participants adjust to the equipment. None of the patients or the controls had previously performed a VO_2max_ test. The Jäger Oxycon Champion spirometric analyzer (Erich Jäger GmBH & Co, Würzburg, Germany) and the software program Vmax 29 (SensorMedics, Anaheim, CA, USA) were used to measure oxygen consumption (VO_2_), ventilation (V_E_) and respiratory exchange ratio (RER). These instruments were calibrated at the beginning of each testing day, and has shown high accuracy and stability [44]. Heart rate was monitored by Polar RS100 (Polar Electro Oy, Kempele Finland). Participants rated the intensity of the work load using Borg’s rating of perceived exertion (RPE) 6–20 scale. Two experienced test leaders carried out the tests. VO_2max_ was terminated when (1) the VO_2_ measures leveled off and participants were unable to maintain work load, (2) RER >1.05, (3) heart rate > 90% of age predicted maximum, and (4) Borg’s RPE ≥ 18. 

#### 2.3.4. Muscular Strength

One repetition maximum (1RM) was tested in the following order and apparatus (Technogym, Italy): Lower body (leg press) and upper body (seated chest press). Correct technique for leg press was 90° in the knee, and heels, back and glutea in touch with surface. Correct chest press technique was to have back and glutea against seat, and full extension of the elbow. Four experienced test leaders were carefully instructed about the standardized procedures. All participants performed the 1RM tests after the VO_2max_ testing with a 15–20 minutes break in between. The 1RM testing procedure was in accordance with previous descriptions [45]: 10 × 50% of 1RM, 1-minute break, 5 × 75% of 1RM, 1-minute break and then 1RM tries with 3-minutes breaks between each try. No participants needed more than four tries at 1RM. The terminated 1RM was the weight (kg) of the last approved repetition with correct technique and performance. 

#### 2.3.5. Body Composition and BMD

Dual x-ray absorptiometry (DXA, Prodigy, GE Lunar, Chalfont St. Giles, UK) was used to measure body composition and BMD. The same operator performed all scanning and analyses, and participants were instructed not to eat or drink later than two hours before the test. BMD measurement areas were lumbar spine (L2–L4), femur neck and total body. Body composition assessments reported are total body weight (kg), lean body mass (kg) and body fat (%). The DXA has been found as an accurate assessment method for both BMD and body composition, with a standard error estimation of ±1–2% [30]. The cut off point for excessive body fat percentage for adult was set to 33%, based on healthy body fat ranges suggested by Gallagher *et al.* [29]. Osteopenia was classified as z-score between −1.0 and −2.5, and osteoporosis was classified as t-score −2.5 or less according to the recommendations for premenopausal women [46].

### 2.4. Statistics

The software SPSS version 15.0 was used to perform statistical analyses. Frequency, mean and standard deviation, paired sample and independent sample t-test and Pearson’s r were performed. Significance level was set to 0.05. 

## 3. Results

Seven patients with BMI < 18.5 (range 14.6–18.4) and 22 patients with BMI ≥ 18.5 (range 19.5–29.3) participated. Four of the patients with BMI ≥ 18.5 were classified as overweight (BMI > 25), but due to the low sample size they are grouped together with the normal weight patients. Table 1 shows no differences between patients with BMI <18.5 and ≥18.5 in EDE and EDI scores, duration of eating disorder, objectively monitored weekly physical activity level at admission and self-reported physical activity level for the four weeks prior to admission. The patients with BMI < 18.5 had longer treatment period at Modum Bad (*p* < 0.01). During treatment there was a significant reduction in ED psychopathology assessed through EDE global score and EDE total score (*p* < 0.001) [47].

**Table 1:** Descriptive data. Values are shown in mean (SD) or n (%).

	BMI < 18.5	BMI ≥ 18.5	*t*-value
	(n = 7)	(n = 22)	
Age (years)	31.9 (9.4)	30.6 (9.0)	0.3
Height (cm)	164.4 (6.8)	169.0 (5.9)	1.9
Total body weight (kg)	46.0 (6.6)	65.4 (9.8)	5.2 ***
Lean body mass (kg)	37.2 (3.6)	43.2 (4.8)	3.3 **
Body fat (%)	15.9 (9.2)	30.5 (8.9)	4.1 ***
BMI (kg/m^2^)	16.8 (1.5)	22.6 (2.6)	5.6 ***
Weekly PA (counts/min)	727.3 (372.4)	595.0 (196.5)	1.3
PA four weeks prior to admission (min/week)	236.7 (505.4)	247.8 (349.0)	0.07
**DSM-IV diagnosis:**			
Anorexia Nervosa	3 (43)	0 (0)	
Bulimia Nervosa	1 (14)	11 (50)	
ED not otherwise specified	3 (43)	11 (50)	
ED duration (years)	16.7 (9.4)	14.8 (7.8)	0.5
EDE global score	4.5 (0.5)	4.5 (0.8)	0.01
Treatment duration (weeks)	20.7 (5.0)	14.2 (4.9)	3.3 **

BMI: body mass index; ED: eating disorder; PA: physical activity; ** *p* < 0.01; *** *p* < 0.001.

### 3.1. Changes in Physical Fitness and BMD

There was an increase in lower body muscular strength among patients with BMI < 18.5, and upper body muscular strength among patients with BMI ≥ 18.5. Ventilation (l/min) increased from admission to discharge, but no significant changes in aerobic fitness were observed (Table 2). Fourteen of the 29 patients were classified as having osteopenia, and one patient had osteoporosis at admission. Three of the 14 patients with osteopenia reported use of oral contraceptives at admission. The patient with osteoporosis reported use of medication (Calcium supplement 1000 mg per day), but not oral contraceptives, at admission. Two patients with BMI < 18.5 went from osteopenia at admission to normal score at discharge. The other patients’ classifications remained unaltered. Mean bone mineral content and femur neck BMD were unaltered in both patient groups, whereas lumbar spine BMD increased in patients with BMI < 18.5 (Table 2).

### 3.2. Changes in Body Composition

**Figure 1:**
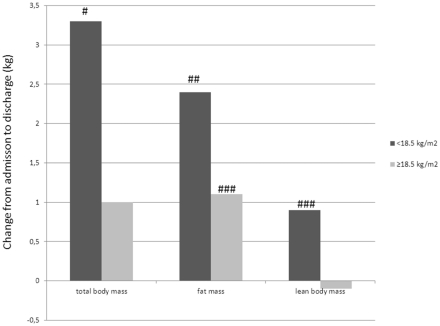
Changes in body mass from admission to discharge. # change is statistically significant (*p* < 0.001); ## change is statistically significant (*p* < 0.01); ### change is statistically significant (*p* < 0.05).

The mean total body mass, fat mass and lean body mass increased among patients with BMI < 18.5 (Figure 1). The mean (SD) BMI increased from 16.8 (1.5) to 18.2 (1.3) kg/m^2^ among the patients with BMI < 18.5 (*p* < 0.01), and from 22.5 (2.6) to 22.9 (2.5) kg/m^2^ among the patients with BMI ≥ 18.5 (*p* < 0.05). 

**Table 2:** Changes in physical fitness and bone health during treatment.

	**BMI < 18.5 (n = 7)**		**BMI >18.5 (n = 22)**		**Diff BMI groups**	
	**Adm**	**Dis**	**Adm**	**Dis**	**Adm**	**Dis**
**Physical fitness**						
VO2max (mL/kg/min)	42.2 (6.1)	43.7 (4.8)	37.9 (9.4)	38.4 (5.8)		
VE (l/min)	71.9 (21.6)	78.7 (24.1) *	90.2 (18.9)	91.2 (18.9)	*	
RER	1.24 (0.09)	1.22 (0.06)	1.22 (0.08)	1.20 (0.08)		
1RM leg press (kg)	60.8 (23.1)	77.1 (24.8) ***	100.8 (31.7)	107.1 (32.3)	**	*
1RM seated chest press (kg)	24.6 (6.6)	34.2 (8.8)	33.3 (10.5)	39.6 (10.3) **		
**Bone health**						
BMC (g)	2123.4 (252.3)	2127.9 (248.3)	2632.1 (402.8)	2657.7 (432.0)	**	**
BMD lumbar spine (g/m^2^)	1.07 (0.14)	1.08 (0.14) *	1.22 (0.16)	1.23 (0.15)	*	*
Z-score lumbar spine	−1.10 (1.13)	−0.97 (1.21) *	0.11 (1.34)	0.22 (1.29) *	*	*
BMD femur neck (g/m^2^)	0.85 (0.13)	0.85 (0.12)	1.02 (0.17)	1.02 (0.17)	*	*
Z-score femur neck	−1.40 (1.08)	−1.28 (1.20)	−0.05 (1.29)	−0.02 (1.28)	*	*

VE: ventilation; RER: respiratory exchange ratio; BMC: bone mineral content; BMD: bone mineral density; Diff: differences between BMI < and ≥ 18.5; * *p* < 0.05; ** *p* < 0.01.

Three of the seven patients with BMI < 18.5 at admission remained underweight at discharge (Table 3). Fat mass, but not lean body mass, increased among patients with BMI ≥ 18.5. As shown in Table 3, none of the patients with BMI < 18.5 and nine of the patients with BMI ≥ 18.5 had excessive amounts of body fat at admission. Three patients reduced body fat percentage from excessive to within normal range from admission to discharge, while one patient increased the body fat percentage to excessive amounts at discharge. The patients with body fat percentage above 40 showed none or low changes in body fat percentage during the treatment period (Table 3). Increased BMI and total body weight, but not body fat percentage, were significantly correlated with increased scores of EDI subscales body dissatisfaction and bulimia, and decreased EDE subscale restraint score (Table 4).

**Table 3:** Changes in BMI, body fat percentage and android/gynoid ratio in each patient during the treatment period.

	Age (years)	Treatment (weeks)	BMI admission	BMI discharge	% change	BF admission	BF discharge	% change	A/G ratio admission	A/G ratio discharge	% change
**BMI < 18.5**											
#1	48.5	22	14.6	17.0	16.4	4.2	14.1	9.9	0.90	0.69	−23.3
#2	33.9	17	16.7	19.0	13.8	5.4	11.5	5.7	0.37	0.51	37.8
#3	28.4	10	17.2	18.0	4.7	20.6	25.5	4.9	0.68	0.82	20.6
#4	20.6	22	18.2	18.7	2.8	25.3	24.2	−0.9	0.68	0.61	−4.4
#5	30.3	25	15.0	16.2	8.0	9.1	17.0	7.9	0.32	0.60	87.5
#6	28.3	24	17.4	18.5	6.3	12.4	13.6	1.2	0.25	0.23	−8.0
#7	42.1	22	18.4	20.2	9.8	22.2	24.7	2.5	0.41	0.43	4.9
**BMI ≥ 18.5**											
#8	21.3	18	19.9	20.8	4.5	25.2	29.9	4.7	0.66	0.68	3.0
#9	23.5	21	21.9	21.7	−0.9	27.4	31.0	3.6	0.88	0.94	6.8
#10	27.3	26	19.9	21.0	5.5	25.5	29.3	3.6	0.81	0.92	13.6
#11	27.0	11	20.3	20.3	0.0	31.9	31.6	−0.3	0.79	0.89	12.7
#12	23.9	21	20.4	21.0	2.9	23.5	26.0	2.9	0.60	0.70	16.7
#13	25.3	20	20.5	22.3	8.8	24.6	32.1	7.5	0.76	0.84	10.3
#14	27.3	21	24.6	24.6	0.0	29.0	31.2	2.2	0.70	0.69	−1.4
#15	38.5	10	20.4	19.4	−4.9	21.7	17.5	−4.2	0.62	0.47	−24.2
#16	39.2	10	24.2	25.0	3.3	42.4	44.6	2.2	0.96	0.94	−2.1
#17	53.8	20	20.8	21.7	4.3	14.3	18.8	4.5	0.47	0.47	0
#18	37.8	13	22.8	22.3	−2.2	35.4	33.8	−1.6	0.93	0.82	−11.8
#19	43.5	13	24.1	24.2	0.4	37.3	37.2	−0.1	1.02	0.97	−4.9
#20	23.1	20	21.5	23.1	7.4	31.6	34.0	2.4	0.63	0.73	15.9
#21	21.5	20	23.4	23.6	0.5	26.6	28.6	2	0.64	0.63	−1.6
#22	22.0	19	23.8	24.0	0.8	36.4	38.9	2.5	0.78	0.73	−6.4
#23	26.4	12	19.8	20.4	3.0	36.1	32.8	−3.3	0.88	0.80	−9.1
#24	26.6	12	20.3	22.6	11.3	30.6	32.0	1.9	0.65	0.67	3.1
#25	37.4	22	19.5	19.9	2.1	13.4	18.6	5.2	0.23	0.30	30.4
#26	18.0	11	25.7	24.0	−6.9	34.1	31.4	−2.7	0.97	0.78	−19.9
#27	27.3	10	26.1	25.7	−1.5	42.2	42.5	0.3	0.90	0.93	3.3
#28	31.6	10	26.4	26.0	−1.5	41.8	41.5	−0.3	1.06	0.99	−6.6
#29	44.1	10	29.3	30.1	2.7	41.8	40.8	−1	1.02	0.98	−3.9

BMI: body mass index; BF: body fat; A/G ratio: android/gynoid ratio.

**Table 4:** Correlations between changes in body composition and ED psychopathology.

	Change total body weight (kg)	Change BMI (kg/m^2^)	Change body fat (%)
**Change EDI**			
Total score	0.28	−0.26	−0.13
Drive for thinness	0.20	−0.11	0.06
Body dissatisfaction	0.49 *	0.44 *	0.26
Bulimia	0.51 **	0.46 *	0.36
Ineffectiveness	0.25	0.22	−0.05
Maturity fears	−0.28	−0.25	−0.14
Perfectionism	0.06	0.02	−0.05
Interpersonal distrust	0.04	0.01	−0.11
Interoceptive awareness	0.14	0.24	0.39
Asceticism	0.17	0.09	−0.06
Social insecurity	0.13	0.13	−0.20
Impulse regulation	0.15	0.08	−0.10
**Change EDE**			
Global score	−0.03	−0.08	−0.12
Restraint	−0.41 *	−0.44 *	−0.31
Eating concern	−0.14	−0.16	−0.15
Weight concern	0.15	0.14	0.06
Shape concern	0.26	0.22	0.13

BMI: body mass index; EDI: eating disorders inventory; EDE: eating disorders examination; * *p* < 0.05; ** *p* < 0.01.

## 4. Discussion

The main findings of this study were the increase in body fat percentage among patients with normal body fat percentage, and that three of the seven patients with BMI <18.5 at admission were still underweight at discharge. Two of these patients had a BMI below 17.5 at discharge, which is the current cut off value for the AN diagnosis [48]. There was a statistically significant increase in total body weight and total fat mass among patients with BMI above as well as below 18.5, however, the net value was a mean increase of 3.3 kg over a mean treatment period of about 16 weeks for the BMI < 18.5 patients. Although this gave an effect size of 1.9, it is far less than reported in other studies [12,34]. A possible explanation for this is that the patients in the studies of Waller *et al.* [12] and Mayer *et al.* [34] had lower mean body weight and BMI compared to the underweight patients of this study. It is important to emphasize that the patients in the current study, although not everyone reached their aimed weight gain, showed significant reduction in ED psychopathology from admission to discharge. As this was the primary treatment goal, it is possible that the weight restoration continues after discharge. 

The weight gain among patients with BMI ≥ 18.5 was on average 0.9 kg, which has no clinical relevance. However, the fat mass increased significantly among the patients who already had a normal body fat percentage, and two patients went from a normal body fat percentage (*i.e.*, 20–32%) at admission to an increased risk of obesity related lifestyle diseases at discharge. Although the main focus during treatment was, and still needs to be, management of ED symptoms and psychopathology, there was an inverse relationship between changes in BMI and total body weight, and changes in e.g., body dissatisfaction and bulimia. In a public health perspective, the negative change of body composition among the normal weight patients is an important issue to address. 

Changes in android/gynoid (A/G) ratio varied among the individuals from an increase of 88% to a decrease of 24%. The android mass refers to visceral fat mass, and the gynoid fat mass refers to fat mass on the hips. An increase in A/G ratio indicates an altered body fat distribution, this has been shown by Mayer *et al.* [34]. Previous studies have found hypercortisolemia among adult, but not adolescent, females with AN, and that these high levels of cortisol can contribute to increased A/G ratio [35].

The increase in muscular strength is in accordance with among others Rigaud *et al.* [13]. Due to the low physical fitness at admission, one of the goals in the physical activity sessions during treatment was to increase both aerobic capacity and muscular strength. The fact that several of the patients had low aerobic capacity despite high levels of vigorous intensity physical activity can possibly be explained by inadequate recovery and low energy availability [8,49]. When the volume of physical activity is reduced and the energy intake is more optimal, the patients should be able to increase in aerobic capacity. However, recovery includes not only energy and nutrient intake, but also sleep and rest. If a person is very psychologically distressed, e.g., during school exam periods or periods with sleep deprivation, the recovery time is prolonged [50]. Thus, in patients with ED, both malnutrition and psychological distress may contribute to prolonged exercise recovery. Physical activity amount (*i.e.*, frequency, duration and intensity) was assessed both objectively and through self-report at admission and discharge. During treatment, the patients kept a physical activity log. In a previous paper, we reported the finding of significantly underreporting of weekly physical activity among inpatients with longstanding eating disorders [2]. In addition, the non-prescribed physical activity both prior to admission and during treatment varied greatly among the patients [2,47]. Although the patients were admitted to an inpatient unit, they were allowed to go outside the hospital for walks etc. The real amount of physical activity could therefore have been higher than reported, and may account for some of the lack of improvements in aerobic capacity.

The increase in lumbar spine BMD among the underweight patients is promising, and in accordance with previous studies having found that weight gain and restoration are important factors for improvement of BMD. The fact that all osteopenic or osteoporotic patients, except two, still had low BMD values at discharge can be due to too short duration of treatment or inadequate treatment approaches. It is possible that other changes regarding the geometry and structure of the bone has occurred, without this being captured by the DXA scanning. Unfortunately, we did not measure hormonal variables such as estrogen and insulin-like growth factor (IGF-1). Physical activity, especially strength training, stimulates the release of growth hormone, which again stimulates the release of IGF-1 [51]. This has been found to be an important predictor of bone microarchitecture independent of body mass in females with AN [52], and future studies should therefore measure changes in hormonal levels when examining changes in BMD among adult underweight females with eating disorders.

### 4.1. Strengths and Limitations

The low sample size is a limitation of this study, and reduces the external validity of the results. DXA can give inaccurate measurements in subjects with very low body weight/body fat mass [53]. None of the patients had BMI less than 15.0, but two had body fat percentages of five or less. It is therefore possible that their results can have been more affected by a measurement error than the normal weight patients. To limit the risk of this bias, the same DXA scanner was used for all measures, and the same test leader carried out the measurements in all patients. The use of DXA, considered as gold standard in measuring body composition, strengthens this study. Direct assessment of aerobic capacity is also a strength, as indirect methods which rely on heart rate measurements and equations, are not reliable in a patient population where bradycardia is a common feature.

### 4.2. Clinical and Scientific Implications

It is interesting that changes in BMI and body weight among all patients are positively correlated with restraint (EDE scale), and body dissatisfaction and bulimia (EDI scale). This indicates that the patients feel worse when BMI is normalized. Future studies therefore need to address whether altered body composition and body weight are associated with higher risk of relapse among normal weight patients. The results also call for a more individual tailoring of the treatment programs, so that patients who need to reduce body fat to more healthy levels can get help with this. The main focus of treatment must be reduction in ED symptoms and psychopathology. Nevertheless, it is a dilemma for treatment units that the patient population is very heterogenic and includes patients who need to increase as well as reduce body weight, body fat percentage, and volume of physical activity. We therefore recommend that approaches towards excessive body fat must be done carefully. For example, focus upon optimalization of physical activity can include reduction of both excessive and sedentary behaviors. In a public health perspective, improvement of physical fitness is very important also for this patient group. Physical activity therefore seems to be an important feature of the treatment. Future studies need to address effects of physical activity and exercise on levels of hormones such as IGF-1 and cortisol among patients with eating disorders. It is possible that physical activity can play a more important role for the enhancement of body composition and BMD than previously assumed among both underweight and normal weight patients with eating disorders.

## 5. Conclusions

The results of this study showed unaltered aerobic capacity, improved muscular strength, and increased body weight and body fat percentage among both underweight and normal weight patients with longstanding ED. Among the underweight patients, there was an increase in lean body mass and lumbar spine BMD. Increased total body mass and BMI were significantly correlated to increased body dissatisfaction, restraint and bulimic symptoms among the patients.
